# Synthesis of BiPO_4_/Bi_2_S_3_ Heterojunction with Enhanced Photocatalytic Activity under Visible-Light Irradiation

**DOI:** 10.1186/s11671-015-1092-z

**Published:** 2015-10-05

**Authors:** Mengna Lu, Guotao Yuan, Zuoshan Wang, Yuyuan Wang, Jun Guo

**Affiliations:** College of Chemistry, Chemical Engineering and Materials Science, Soochow University, Soochow, 215123 China

**Keywords:** BiPO_4_/Bi_2_S_3_, Photocatalytic activity, Hydrothermal method, Heterojunction photocatalyst

## Abstract

BiPO_4_/Bi_2_S_3_ photocatalysts were successfully synthesized by a simple two-step hydrothermal process, which involved the initial formation of BiPO_4_ rod and then the attachment of Bi_2_S_3_ through ion exchange. The as-synthesized products were characterized by X-ray diffraction (XRD), scanning electron microscope (SEM), transmission electron microscopy (TEM), X-ray photoelectron spectroscopy (XPS), and UV-vis diffuse reflectance spectra (UV-vis DRS). It was found that BiPO_4_ was regular rods with smooth surfaces. However, BiPO_4_/Bi_2_S_3_ heterojunction had a rough surface, which could be attributed to the attachment of Bi_2_S_3_ on the surface of BiPO_4_ rods. The BiPO_4_/Bi_2_S_3_ composite exhibited better photocatalytic performance than that of pure BiPO_4_ and Bi_2_S_3_ for the degradation of methylene blue (MB) and Rhodamine B (RhB) under visible light. The enhanced photocatalytic performance could be ascribed to synergistic effect of BiPO_4_/Bi_2_S_3_ heterojunction, in which the attached Bi_2_S_3_ nanoparticles could improve visible-light absorption and the BiPO_4_/Bi_2_S_3_ heterojunction suppressed the recombination of photogenerated electron-hole pairs. Our work suggested that BiPO_4_/Bi_2_S_3_ heterojunction could be a potential photocatalyst under visible light.

## Background

Currently, semiconductor photocatalysts have attracted a lot of interests due to their widely applications for the degradation of organic contaminants [1–4] and generation of hydrogen from water [5]. Generally speaking, a highly efficient photocatalyst must have a wide photoabsorption range, as well as the low recombination rate of photogenerated electron-hole pairs. Therefore, it is also a challenge to develop a new compound with high photocatalytic efficiency under visible light [6–9].

As a potential photocatalyst, BiPO_4_ has recently been extensively studied [10–12]. It has been reported that the photocatalytic activity of BiPO_4_ is strongly dependent on its crystal structure [13] and the monoclinic phase BiPO_4_ showed a better photocatalytic performance than that of P25 for the photodegradation of organic contaminants under UV irradiation [14]. However, BiPO_4_ had a wide band gap of about 3.8 eV and thus can only be excited by UV light to generate electron-hole pairs [11]. In order to improve the visible-light utilization of BiPO_4_, many efforts have been taken. Lin et al. fabricated Ag_3_PO_4_/BiPO_4_ heterojunction with enhanced photocatalytic ability under visible-light irradiation [15]. Duo et al. reported that BiPO_4_/BiOCl heterojunction also had enhanced photocatalytic activity [16]. Li et al. found that BiPO_4_/g-C_3_N_4_ heterojunction could efficiently respond to visible-light irradiation [17]. Besides, Zhang et al. reported that BiPO_4_/reduced graphene oxide composites with specific surface areas had better photocatalytic activity for the degradation of MB [18]. Whereas, coupling of BiPO_4_ with other semiconductors is still meaningful for improving light absorption in the visible spectrum and suppressing the recombination of the photogenerated electron-hole pairs more effectively.

Bi_2_S_3_, a small band gap semiconductor (1.3 eV), has a high photoabsorption coefficient [19–21]. Hence, it can usually be used as a potential visible-light photocatalyst through combination from other semiconductors to improve light absorption and separation efficiency of photogenerated electron-hole pairs, such as CdS/Bi_2_S_3_ [22], BiVO_4_/Bi_2_S_3_ [23], Bi_2_S_3_/BiOBr [24], and so on.

In this study, we reported the preparation of a novel BiPO_4_/Bi_2_S_3_ heterostructure and their photocatalytic properties were evaluated by the degradation of MB and RhB under visible light. As expected, the as-prepared BiPO_4_/Bi_2_S_3_ heterojunction exhibited enhanced visible-light photocatalytic activity and a possible mechanism was presented.

## Methods

### Materials and Preparation

All reagents were of analytical purity (Sinopharm Chemical reagent Co., Ltd., China) and used without further purification.

### Synthesis of BiPO_4_

BiPO_4_ was prepared by a facile hydrothermal method. Firstly, 0.5 g of PVP was dissolved in a beaker with deionized water (50 mL) under stirring. Secondly, Bi(NO_3_)_3_ · 5H_2_O and NaH_2_PO_4_ · 12H_2_O (molar radio of 1:1) were added into the solution. After the pH of the reaction system was adjusted to 3 by HNO_3_, the solution was transferred into a 100-mL Teflon-lined stainless steel autoclave and heated at 180 °C for 24 h. When the system cooled down to room temperature naturally, the resulting product was harvested and washed with deionized water and absolute alcohol for several times. Finally, the as-prepared products were dried at 60 °C for 12 h.

### Synthesis of BiPO_4_/Bi_2_S_3_ Photocatalyst

The BiPO_4_/Bi_2_S_3_ photocatalyst was prepared through an in situ ion exchange process. Typically, 0.1 g of PVP was dissolved in 50 mL of ethylene glycol, followed by the addition of 0.456 g of BiPO_4_ under stirring to achieve suspension. Then, a certain amount of thiourea (the amount of thiourea was 0.086, 0.172, and 0.573 g, and they are named as BB-1, BB-2, and BB-3, respectively.) was added into above suspension and the solution was transferred into a 100-mL Teflon-lined stainless steel autoclave, which was sealed and maintained at 140 °C for 3 h. After the autoclave was cooled to room temperature naturally, the precipitates were collected and washed with water and ethanol several times. The BiPO_4_/Bi_2_S_3_ products were dried at 60 °C for 12 h. For comparison, pure Bi_2_S_3_ was prepared through hydrothermal method according to the literature [25].

### Characterization of the As-prepared Samples

The phase of the samples was measured by XRD (D/Max-ШC, Shimadzu) using an X-ray diffractometer with Cu Kα radiation. The morphology was analyzed by SEM on Hitachi S-4600 and TEM (FEI Tecnai G20). UV-vis DRS was tested on a Shimadzu UV240 UV-vis spectrophotometer with BaSO_4_ as a reference material. The elemental composition of the samples was analyzed by X-ray photoelectron spectrometer (XPS, USA Thermo ESCALAB 250).

### Photocatalytic Activity

The photocatalytic performance of BiPO_4_/Bi_2_S_3_ heterojunction photocatalyst was evaluated by the degradation of MB and RhB under visible light. In each experiment, 50 mg of different photocatalysts were added into 100 mL of MB or RhB solution (10 mg/L) in a reactor. Before irradiation, the mixture was magnetically stirred for 30 min in the dark to achieve the adsorption/desorption equilibrium between dye and photocatalysts. Then, the solution was irradiated by visible light under continuous stirring. At a defined time interval, about 3 mL of solution was extracted from the reactors and then centrifuged to remove catalysts before analysis. Finally, MB (RhB) solution was analyzed through a UV-vis spectrophotometer. The degradation rate could be obtained through the formula [26]: *η* = *C*_*i*_/*C*_0_ × 100 %, where *C*_*i*_ was the absorbance of MB (RhB) which was measured every 30 min, and *C*_0_ was the absorbance of MB (RhB) before light up.

## Results and Discussion

### Phase and Crystal Structure Analysis

**Fig. 1:**
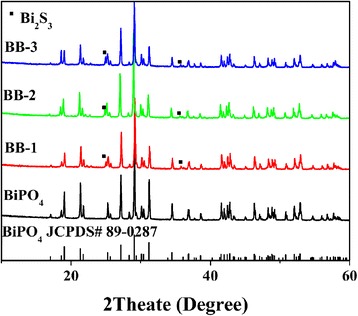
XRD patterns of different as-prepared samples

Figure 1 shows the XRD patterns of BiPO_4_ and BiPO_4_/Bi_2_S_3_ heterojunction with different Bi_2_S_3_ contents. In the pure BiPO_4,_ all the diffraction peaks are well matched with the monoclinic phase of BiPO_4_ (JCPDS File No. 89–0287), indicating that the as-prepared BiPO_4_ has the high purity. On the other hand, the BiPO_4_/Bi_2_S_3_ composites exhibit a mixture of two crystalline phases. One can be identified as BiPO_4_, and the others originate from rutile Bi_2_S_3_ [25]. Furthermore, the intensities of corresponding to diffraction peaks of Bi_2_S_3_ gradually strengthen along with the increase of the Bi_2_S_3_ content, while those of BiPO_4_ simultaneously weaken. No other characteristic peaks of impurity are detected, suggesting that BiPO_4_/Bi_2_S_3_ composites are only composed of BiPO_4_ and Bi_2_S_3_ phases.

**Fig. 2:**
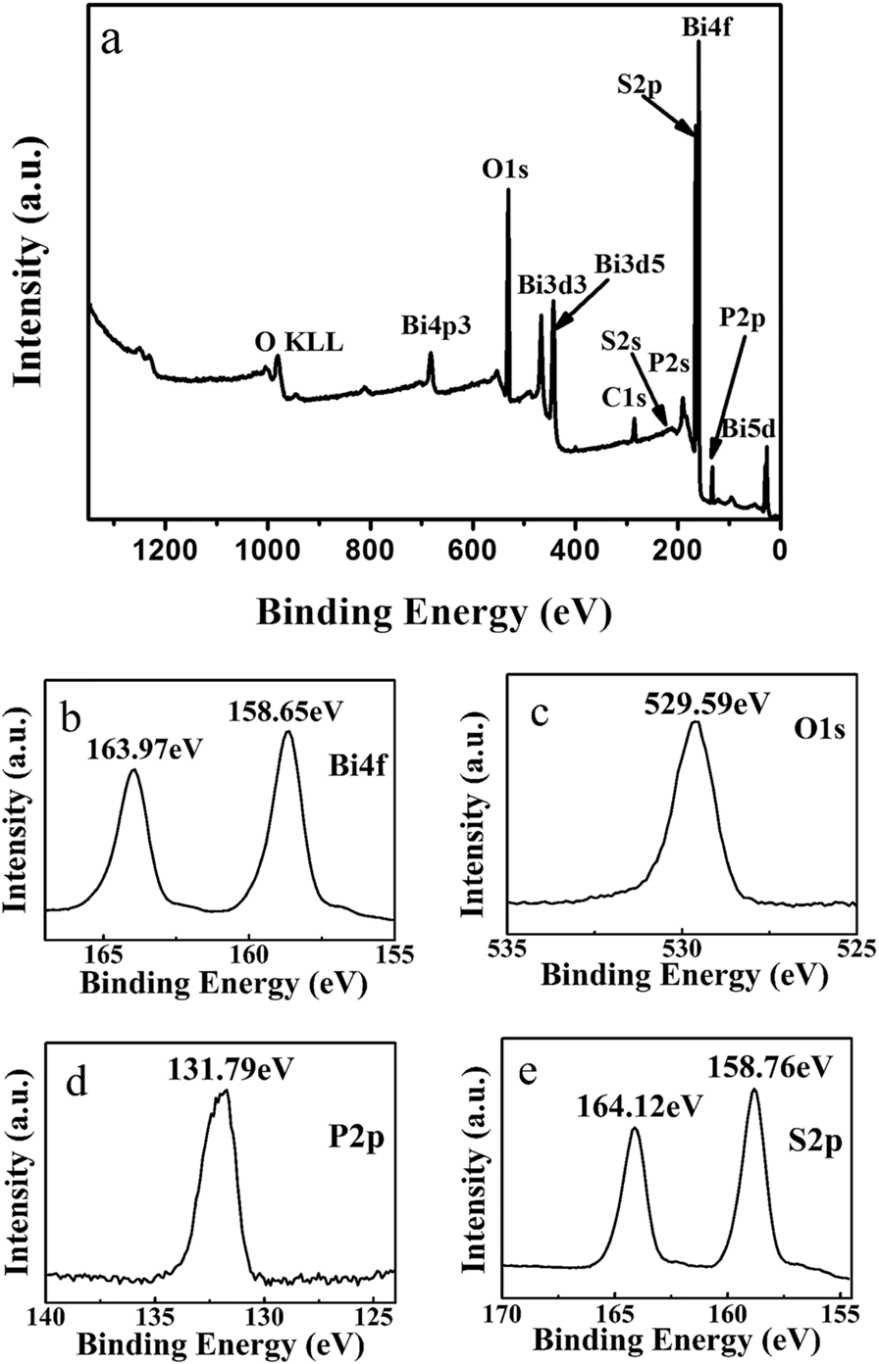
The XPS survey spectra of BB-2 (**a**) and high-resolution XPS spectra of Bi 4f (**b**), O 1 s (**c**), P 2p (**d**) and S 2p (**e**)

The surface chemical composition of BB-2 is analyzed by XPS and the results are shown in Fig. 2. The XPS survey spectrum (Fig. 2a) shows that BB-2 contains Bi, P, S, and O elements, which is consistent to XRD results. Besides, C 1 s peak is also seen in XPS survey spectrum, which can be attributed to adventitious hydrocarbon from instrument. Two peaks appear at 163.97 and 158.65 eV in Fig. 2b, which are corresponding to Bi 4f_5/2_ and Bi 4f_7/2_ peaks of Bi^3+^, respectively [27]. In Fig. 2c, O 1 s peak appeared at 529.59 eV, in which it can be attributed to lattice oxygen in crystalline BiPO_4_ [28]. In Fig. 2d, the P 2p XPS peak appeared at 131.79 eV, suggesting that P exists in the oxidation of P^5+^. On the other hand, the binding energies of 164.12 and 158.76 eV are attributed to S 2p peaks (Fig. 2e), which prove the existence of S^2−^ [29].

### Morphology Analysis

**Fig. 3:**
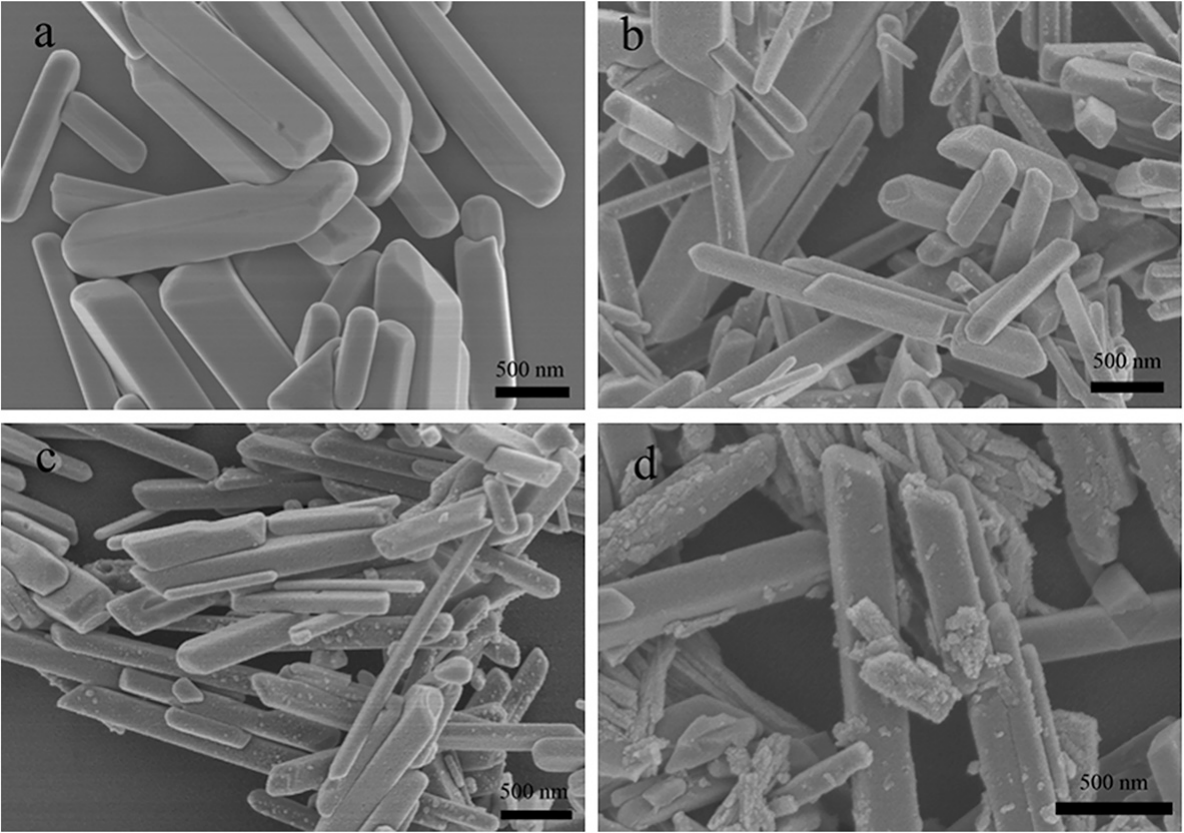
SEM images of pure BiPO_4_ (**a**), BB-1 (**b**), BB-2 (**c**) and BB-3 (**d**)

Figure 3 shows the SEM images of BiPO_4_ and BiPO_4_/Bi_2_S_3_ composites. It can be seen from Fig. 3a that pure BiPO_4_ shows regular rod shape with diameter of 200–400 nm and the length of 500–2000 nm. It should be noted that these rods have smooth surfaces. Figure 3b–d shows the SEM images of different BiPO_4_/Bi_2_S_3_ composites. Compared with pure BiPO_4_, the surfaces of BiPO_4_/Bi_2_S_3_ composites become rough. Furthermore, with the increasing amount of additive thiourea, more Bi_2_S_3_ nanoparticles can be observed on the surface of BiPO_4_ rods gradually, which is also consistent to XRD results.

**Fig. 4:**
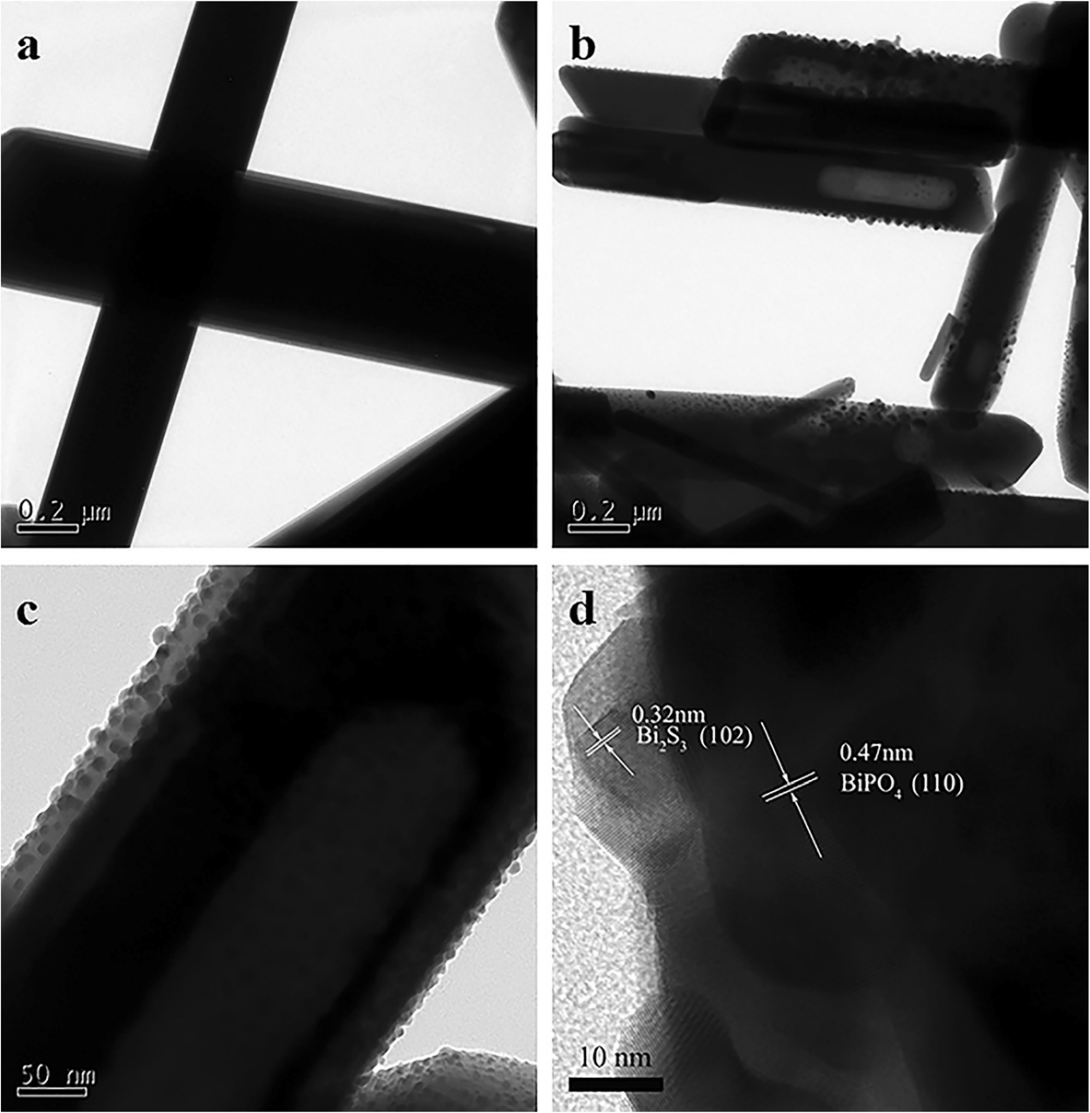
TEM images of BiPO_4_ (**a**), BB-2 (**b**, **c**), and HRTEM image of BB-2 (**d**)

TEM and HRTEM images are shown in Fig. 4, which display identified results as those of SEM analysis. From Fig. 4a, one can see that pure BiPO_4_ are regular rods with a smooth surface. While BiPO_4_/Bi_2_S_3_ heterojunction shows a rough surface, suggesting the successful attachment of Bi_2_S_3_ on the surface of BiPO_4_ rods. Furthermore, the lattice spacings can be clearly seen in the corresponding HRTEM image (Fig. 4d). The fringe spacing of 0.47 nm is indexed to the (1 1 0) lattice plane of monoclinic BiPO_4_, while 0.32 nm is agreed with the (1 0 2) lattice plane of Bi_2_S_3_. Therefore, it can be summarized that BiPO_4_/Bi_2_S_3_ heterojunction is achieved through a facile ion-exchange method.

### UV-vis Analysis

**Fig. 5:**
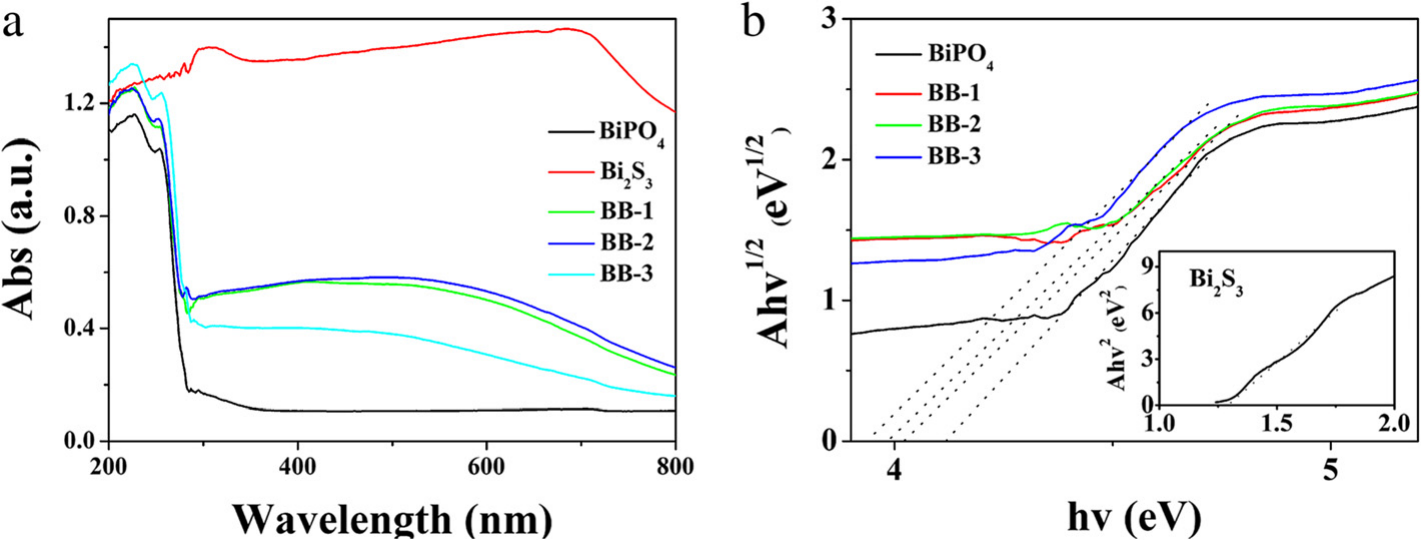
**a** UV-vis DRS of BiPO_4_, Bi_2_S_3_, and BiPO_4_/Bi_2_S_3_ composites and **b** the plotting of (αhν)^n/2^ vs. hν

Figure 5a shows UV-vis DRS of as-prepared BiPO_4_, Bi_2_S_3_, and BiPO_4_/Bi_2_S_3_ composites. It reveals that BiPO_4_/Bi_2_S_3_ composites have a stronger absorption than that of BiPO_4_ in visible light. The band gap energy can be achieved through the formula [30, 31]. Besides, according to the literature, *n* values of BiPO_4_ and Bi_2_S_3_ are 4 [32] and 1 [33], respectively. Therefore, as is shown in Fig. 5b, *E*_*g*_ of BiPO_4_ and Bi_2_S_3_ can be calculated as 4.08 and 1.30 eV. Moreover, *E*_*g*_ of BB-1, BB-2, and BB-3 are 4.01, 3.93, and 3.81 eV, respectively. Besides, Bi_2_S_3_ displays quantum size effect, which may influence the band gap, the position of both CB and VB band. Besides, the band gap shift relative to the bulk can be calculated by the following formula [34, 35]:

$$ \varDelta {E}_g(R)=\frac{h^2}{8{m}_o{R}^2}\left(\frac{1}{{m_e}^{\ast }}+\frac{1}{{m_h}^{\ast }}\right), $$

in which ∆*E*_*g*_ (*R*) is the band gap shift, *h* is the Planck’s constant, and *R* is the crystal radius. Besides, *m*_*o*_ is electron mass and *m*_e_^*^ and *m*_h_^*^ are the effective masses of electrons and holes, respectively. Then, the size of Bi_2_S_3_ nanoparticles attached on the surface of BiPO_4_ rods can be calculated as 2.68, 2.72, and 2.78 nm, respectively, which is much smaller than Bohr excitation radius of 24 nm. Therefore, quantum size confinement can be observed obviously, which influences the band gap, the position of both CB and VB band, etc. These results also support the enhancement of photocatalytic activity.

### Photocatalytic Activity of Different Samples

**Fig. 6:**
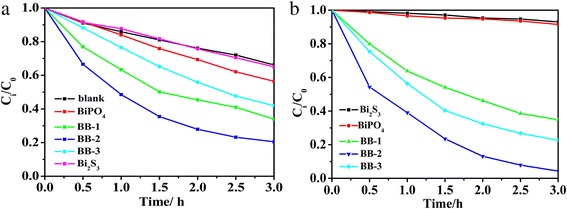
**a** Photodegradation rate of MB under visible-light irradiation with different samples, **b** photodegradation rate of RhB under visible-light irradiation with different samples

The photocatalytic performance of BiPO_4_/Bi_2_S_3_ heterojunction was assessed by photodegradation of MB under visible-light irradiation (Fig. 6a). It can be seen that pure BiPO_4_ shows poor photocatalytic ability in degrading MB (40 %). Interestingly, the coupling of BiPO_4_ with Bi_2_S_3_ leads to notable enhancement MB photodegradation. The MB removal rates are about 50, 80, and 60 %, respectively. Meantime, RhB here is also employed as an organic pollutant to further confirm the photodegradation activity of BiPO_4_/Bi_2_S_3_ heterojunction. As shown in Fig. 6b, BiPO_4_/Bi_2_S_3_ composites show better photocatalytic activity in the degradation of RhB than that of pure BiPO_4_ and the best photocatalytic property was achieved for BB-2 sample. The enhanced visible-light-driven activity of the heterostructure must be attributed to the synergistic effect between BiPO_4_ and Bi_2_S_3_. What is more, the quantum size confinement of Bi_2_S_3_ in the visible spectrum also leads to the enhancement of photocatalytic activity. However, the excess Bi_2_S_3_ content in BiPO_4_/Bi_2_S_3_ composite will cause their photocatalytic performance to decrease (BB-3). It may be attributed to these reasons: one is reduction of active sites due to the excess Bi_2_S_3_ nanoparticles on the surface BiPO_4_ rod [36]. The other is that excessive narrow band gap Bi_2_S_3_ may lower the separation efficiency of electron-hole pairs and further inhibit the photocatalytic activity [37].

### Possible Photocatalytic Mechanism

**Fig. 7:**
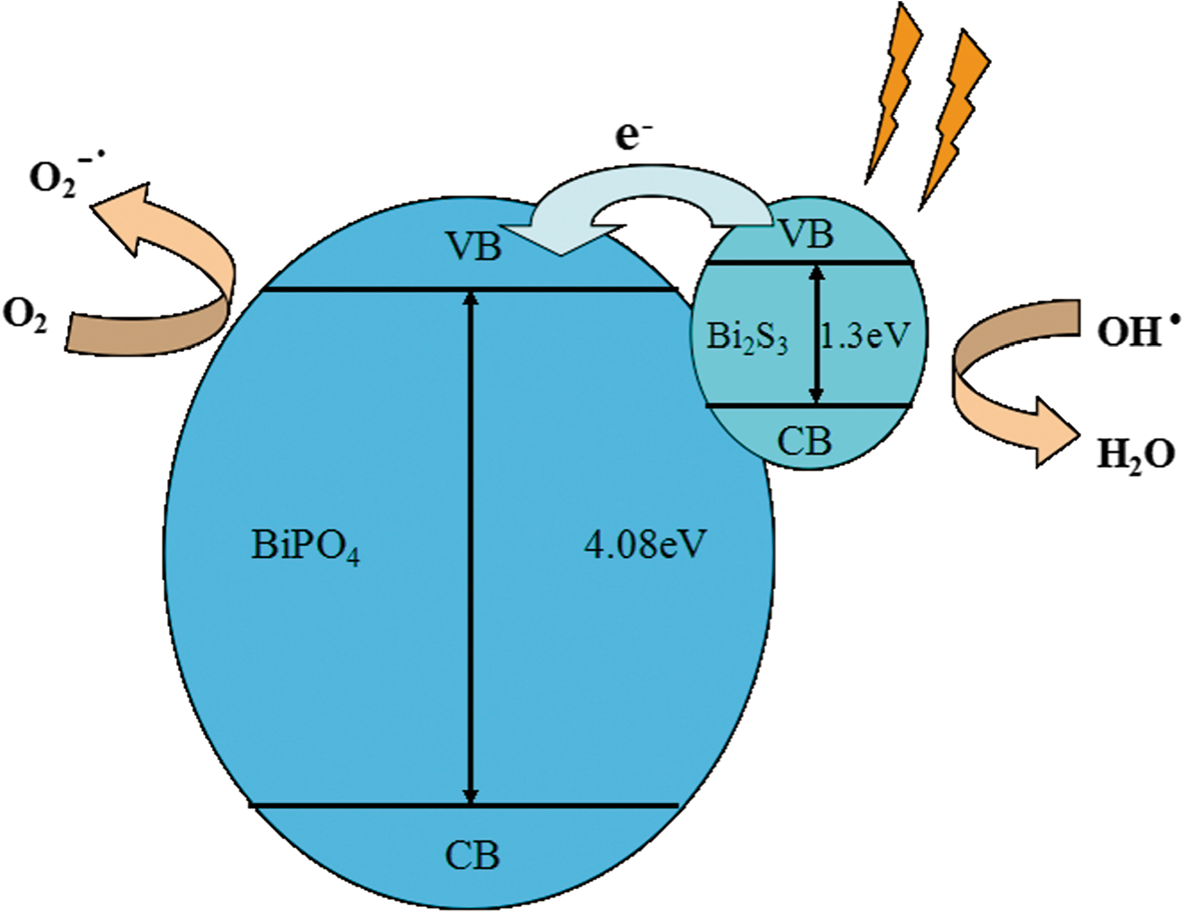
Schematic illustration of possible electrons and hole transfer mechanism of BiPO_4_/Bi_2_S_3_ heterostructure

The band positions of BiPO_4_ and Bi_2_S_3_ are evaluated based on the equation [38]. Hence, the valence band and conduction band edge potential (*E*_VB_ and *E*_CB_) of BiPO_4_ and Bi_2_S_3_ are 4.39 eV, 0.31 eV and 1.43 eV, 0.13 eV, respectively. Therefore, the possible mechanism is shown in Fig. 7. Bi_2_S_3_ nanoparticles absorb the visible light and give rise to electron-hole pairs. The photo-excited electrons in Bi_2_S_3_ CB will transfer to BiPO_4_ rods and holes are left in Bi_2_S_3_ VB, which will decrease recombination rate of photogenerated charge carriers. The electrons in BiPO_4_ CB can rapidly adsorb O_2_ to form O_2_^−•^, while the holes can interact with the absorbed H_2_O to achieve hydroxyl radicals. After then, O_2_^−•^ and OH• with strong oxidizability can decompose MB (RhB) to generate CO_2_ and H_2_O. Moreover, BiPO_4_/Bi_2_S_3_ heterojunction photocatalysts have a stronger and wider absorption in visible light, which is beneficial to photocatalytic activity.

## Conclusions

In summary, we have synthesized the BiPO_4_/Bi_2_S_3_ heterojunction with a facile two-step hydrothermal method. Bi_2_S_3_ nanoparticles can be in situ formed on the surface of BiPO_4_ rods through ion exchange. As the quantum size confinement of Bi_2_S_3_ in the visible spectrum, it can be used as photosensitizer. When BiPO_4_ rods are modified with Bi_2_S_3_, the separation of electron-hole pairs could be accelerated and the photoabsorption could be promoted as well. These directly led to the enhancement of photocatalytic activity for the degradation of MB (RhB) under visible-light irradiation, and BB-2 sample exhibits the best photocatalytic property. Degradation rate of MB under visible-light irradiation with BB-2 could reach to 80 % in 3 h, double that of pure BiPO_4_. Besides, degradation rate of RhB could reach to 99.6 % in 3 h, while it only degraded for 8 % by pure BiPO_4._
